# Supplementary material for: YgfB increases β-lactam resistance in Pseudomonas aeruginosa by counteracting AlpA-mediated ampDh3 expression
Source: Commun Biol. 2023 Mar 10;6:254. doi: 10.1038/s42003-023-04609-4 (PMC9998450; doi:10.1038/s42003-023-04609-4)
Supplement: Supplementary file 1 — Supplementary Information [file 42003_2023_4609_MOESM1_ESM.pdf]

Supplementary information

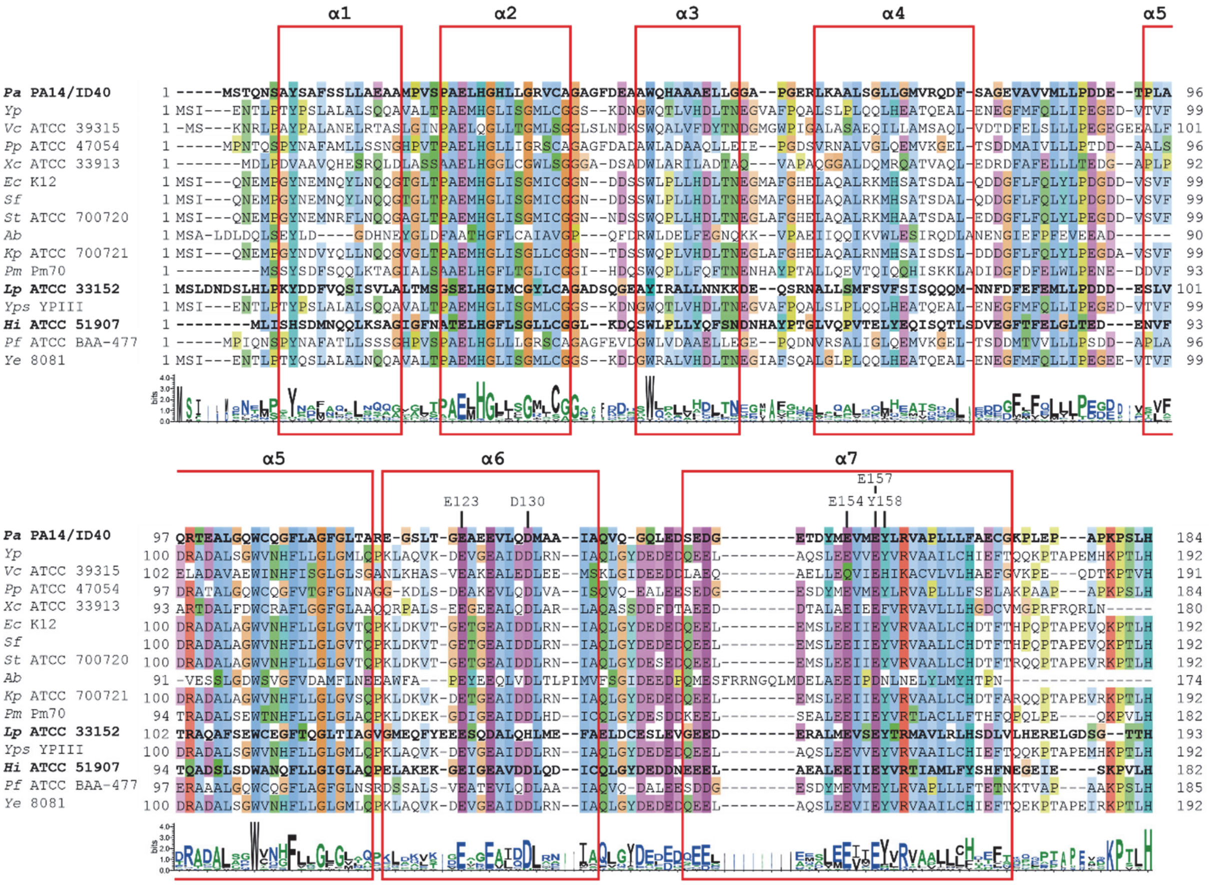

Supplementary Fig. 1: Conservation of YgfB in different species. Sequences of YgfB proteins were aligned using clustalΩ[1, 2] and color coded according to conservation with clustalX[3]. The secondary structure elements as deduced from the crystal structure of the *Haemophilus influenzae (Hi)* protein (pdb ID: 1izm) are indicated as red boxes. Conserved residues in the dimerization interface of *Hi* YgfB[4] are labeled above the alignment. The sequences of the *Pseudomonas aeruginosa* (*Pa*) protein as well as the sequences of the crystal structures of the *Legionella pneumophila* (*Lp*, pdb ID: 4gyt) and *Hi* proteins are highlighted in bold. A sequence logo generated with WebLogo 3[5, 6] is shown below the sequence alignment to highlight conserved residues. The sequence alignment view was prepared with Jalview[7] UniProt accession numbers and species names are listed in **Supplementary Table 1**.

Supplementary Table 1: UniProt accession numbers of the protein sequences aligned in Supplementary Fig. 1.

Species	Name	Accession
Acinetobacter baumannii (Ab)	YgfB/YecA family protein	V5VAK1
Escherichia coli (strain K12) (Ec K12)	UPF0149 protein YgfB	P0A8C4
Haemophilus influenzae (strain ATCC 51907 / DSM 11121 / KW20 / Rd) (Hi ATCC 51907)	UPF0149 protein HI_0817	P44882
Klebsiella pneumoniae subsp. pneumoniae (strain ATCC 700721 / MGH 78578) (Kp ATCC 700721)	UPF0149 protein KPN78578_32810	A6TDS1
Legionella pneumophila subsp. pneumophila (strain Philadelphia 1 / ATCC 33152 / DSM 7513) (Lp ATCC 33152)	lpg0076 protein	Q5ZZD5
Pasteurella multocida (strain Pm70) (Pm Pm70)	UPF0149 protein PM1723	Q9CKA2
Pseudomonas aeruginosa (strain UCBPP-PA14) (Pa PA14/ID40)	UPF0149 protein PA14_69010	Q02ED9
Pseudomonas fluorescens (strain ATCC BAA-477 / NRRL B-23932 / Pf-5) (Pf ATCC BAA-447)	UPF0149 protein PFL_5969	Q4K406
Pseudomonas putida (strain ATCC 47054 / DSM 6125 / NCIMB 11950 / KT2440) (Pp ATCC 47054)	UPF0149 protein PP_5201	Q88CI0
Salmonella typhimurium (strain LT2 / SGSC1412 / ATCC 700720) (St ATCC 700720)	UPF0149 protein YgfB	Q8ZM71
Shigella flexneri (Sf)	UPF0149 protein YgfB	P0A8C7
Vibrio cholerae serotype O1 (strain ATCC 39315 / El Tor Inaba N16961) (Vc ATCC 39915)	UPF0149 protein VC_2476	Q9KP97
Xanthomonas campestris pv. campestris (strain ATCC 33913 / DSM 3586 / NCPPB 528 / LMG 568 / P 25) (Xc ATCC 33913)	UPF0149 protein XCC3260	Q8P5S6
Yersinia enterocolitica serotype O:8 / biotype 1B (strain NCTC 13174 / 8081) (Ye 8081)	UPF0149 protein YE3397	A1JPP0
Yersinia pestis (Yp)	UPF0149 protein PO0911/y3298/YP_3608	Q8ZHI2
Yersinia pseudotuberculosis serotype O:3 (strain YPIII) (Yps)	UPF0149 protein YPK_0862	B1JNS1

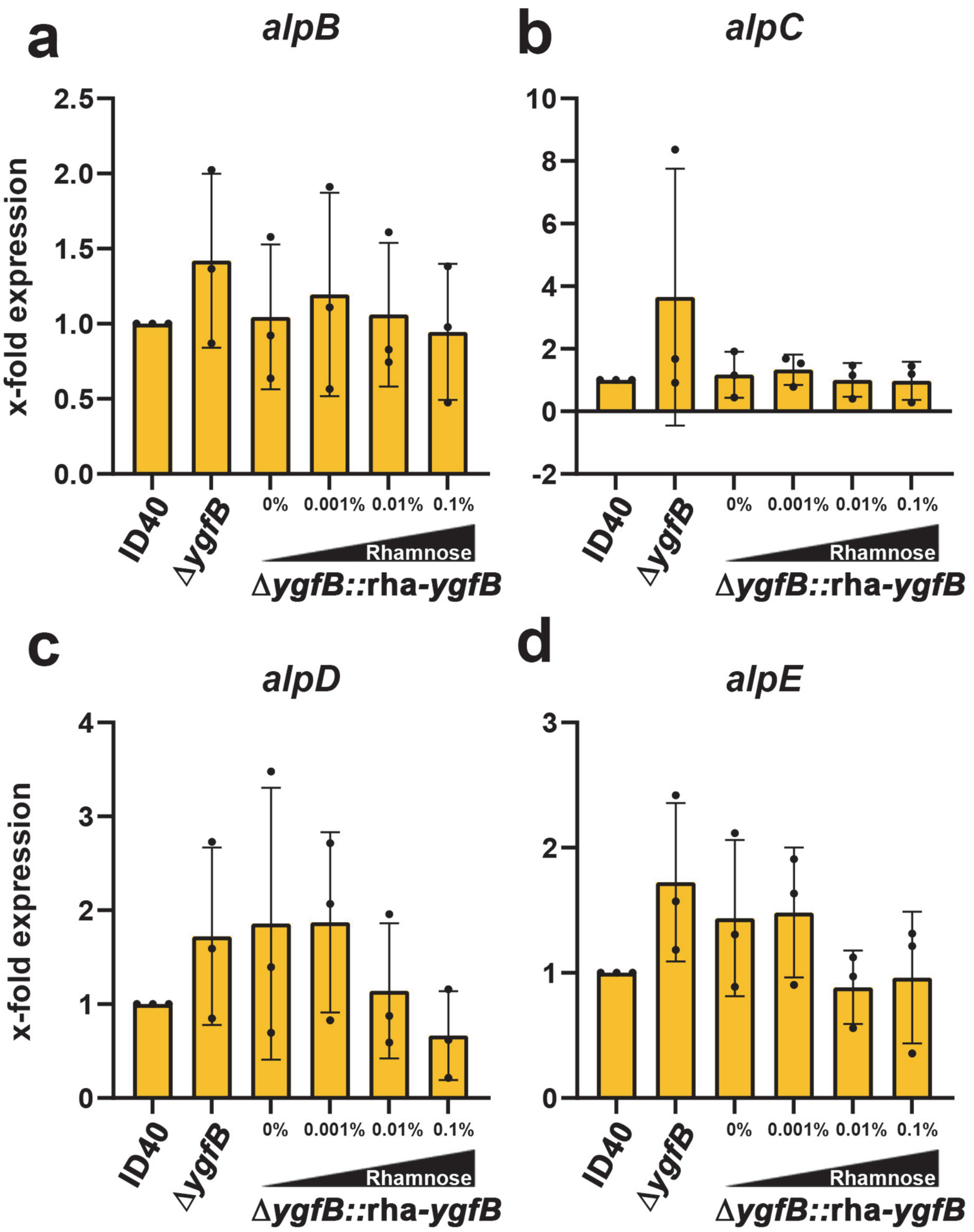

Supplementary Fig. 2: Gene expression of the *alpBCDE* cluster. ID40, ID40Δ*ygfB,* ID40Δ*ygfB*::rha-*ygfB* were grown with the indicated concentrations of rhamnose in LB medium at 37°C to induce *ygfB* expression. After 3h of growth mRNA was isolated and used for RT-qPCR. Data depict mean and SD for x-fold expression of (a) *alpB*, (b) *alpC*, (c) *alpD* and (d) *alpE* compared to ID40 of *n*=3 independent experiments. Differences between conditions were non-significant in one-way ANOVA analysis. Source data are shown in **Supplementary Data 10**.

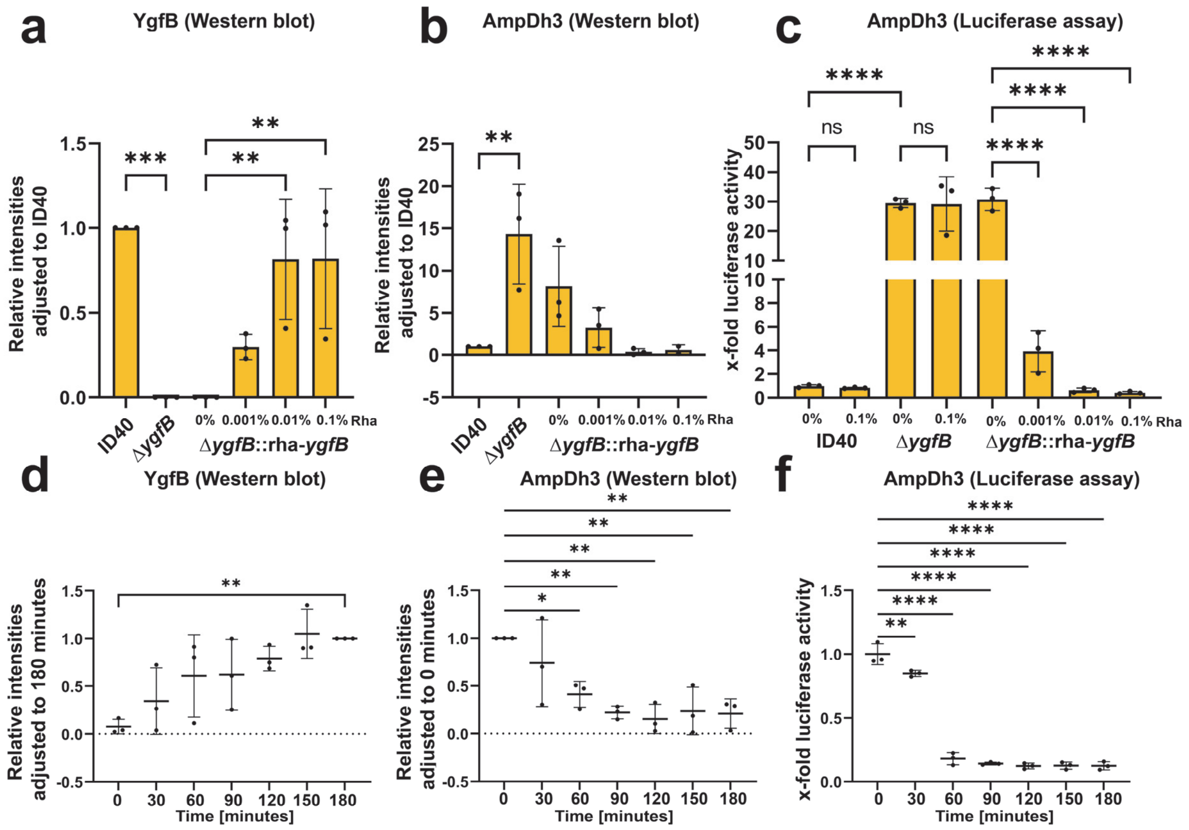

Supplementary Fig. 3: Semiquantification of YgfB and AmpDh3 based on WB analyses and of AmpDh3-HiBiT by luciferase assay.

Western blots shown in **Fig. 1g** (a and b) and **Fig. 2b** (d and e) were quantified using Image J. Bands for YgfB and AmpDh3 were normalized to RpoB. (a) Quantification of YgfB as shown in Fig. 1g and two more replicates, (b) quantification of AmpDh3 as shown in Fig. 1g and two more replicates, (d) quantification of YgfB as shown in Fig. 2b and two more replicates and (e) quantification of AmpDh3 as shown in Fig. 2b and two more replicates. In (c and f) experiments are shown which were performed with the same strains and under the same conditions as the WB analyses but the cells were lysed with a lysis buffer. In this case (c) corresponds to the experimental setup of Fig. 1g and (f) to the experimental setup of Fig. 2b. The lysates were incubated with LgBiT and furimazine to measure relative AmpDh3-HiBiT levels as luciferase activity in a Tecan reader. Of note: in (c) ID40 and Δ*ygfB* strains were in addition treated with 0.1% rhamnose to demonstrate that rhamnose has no impact on AmpDh3 levels. Data depict mean and SD of *n*=2-3 individual experiments or Western blot replicates respectively. Statistical analyses were performed for WT vs. Δ*ygfB* with or without addition of 0.1% rhamnose and the *ygfB* complemented strain without vs. with rhamnose as well as time point zero vs. all other time points. (Asterisks indicate significant differences, ns: not significant, *p<0.05, **p<0.01,***p<0.001, ****p<0.0001, in (d) comparing to timepoint 180 minutes and in (e) comparing to timepoint 0 minutes, one-way ANOVA, Šídák's multiple comparisons). Source data are shown in **Supplementary Data 11.**

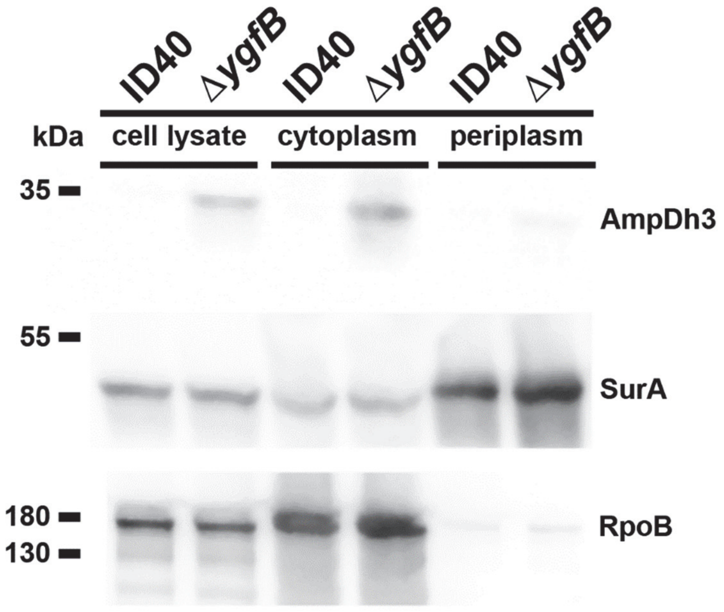

Supplementary Fig. 4: Localization of AmpDh3. Subcellular fractionation using the strains ID40::*ampDh3*-HiBiT and ID40Δ*ygfB::ampDh3*-HiBiT was performed as described in the methods section. Western blots were performed for AmpDh3-HiBiT, SurA and RpoB. To separately detect SurA, RpoB and AmpDh3-HiBiT on the same blot, the membrane has been cut apart horizontally at 70 kDa and at 40 kDa and each piece incubated with the respective antibody or NanoGlo HiBiT Blotting System.

Supplementary Table 2. Fractional inhibitory concentration index (FIC-I). FIC-I ≤ 0.5, synergistic effect, 0.5-1 additive, >1 indifferent.

	CAZ + CIP			PIP + CIP		
**Strain**	**FIC_{CAZ}**	**FIC_{CIP}**	**FIC-I**	**FIC_{PIP}**	**FIC_{CIP}**	**FIC-I**
ID40	0.25	0.25	0.5	0.25	0.5	0.75
Δ*ygfB*	0.25	0.25	0.5	0.25	0.25	0.5
Δ*ampDh3*	0.25	0.25	0.5	0.25	0.5	0.75
Δ*ygfB*Δ*ampDh3*	0.25	0.25	0.5	0.25	0.5	0.75
Δ*alpA*	0.125	0.25	0.375	0.0625	0.25	0.3125
Δ*ygfB*Δ*alpA*	0.125	0.25	0.375	0.125	0.25	0.375
Δ*ygfB*::rha-*ygfB* 0% rha	0.25	0.25	0.5	0.125	0.5	0.625
Δ*ygfB*::rha-*ygfB* 0.1% rha	0.25	0.25	0.5	0.25	0.5	0.75
Δ*ampDh3*::rha-*ampDh3* 0% rha	0.125	0.5	0.625	0.25	0.5	0.75
Δ*ampDh3*::rha-ampDh3 0.1% rha	0.015625	0.5	0.515625	0.5	0.5	1
Δ*alpA*::rha-*alpA* 0%rha	0.25	0.25	0.5	0.125	0.5	0.625
Δ*alpA*::rha-*alpA* 0.1%rha	0.25	0.25	0.5	0.25	0.125	0.375

	IMP + CIP			AZT + CIP		
**Strain**	**FIC_{IMP}**	**FIC_{CIP}**	**FIC-I**	**FIC_{AZT}**	**FIC_{CIP}**	**FIC-I**
ID40	0.25	0.5	0.75	0.25	0.25	0.5
Δ*ygfB*	0.0625	0.5	0.5625	0.25	0.25	0.5
Δ*ampDh3*	0.5	0.25	0.75	0.25	0.25	0.5
Δ*ygfB*Δ*ampDh3*	0.25	0.5	0.75	0.03125	0.5	0.53125
Δ*alpA*	0.125	0.5	0.625	0.25	0.25	0.5
Δ*ygfB*Δ*alpA*	0.125	0.5	0.625	0.125	0.125	0.25
Δ*ygfB*::rha-*ygfB* 0% rha	0.125	0.5	0.625	0.25	0.25	0.5
Δ*ygfB*::rha-*ygfB* 0.1% rha	0.25	0.5	0.75	0.5	0.25	0.75
Δ*ampDh3*::rha-*ampDh3* 0% rha	0.25	0.5	0.75	0.5	0.25	0.75
Δ*ampDh3*::rha-ampDh3 0.1% rha	0.03125	0.5	0.53125	0.5	0.25	0.75
Δ*alpA*::rha-*alpA* 0%rha	0.25	0.5	0.75	0.25	0.25	0.5
Δ*alpA*::rha-*alpA* 0.1%rha	0.03125	0.5	0.53125	0.5	0.25	0.75

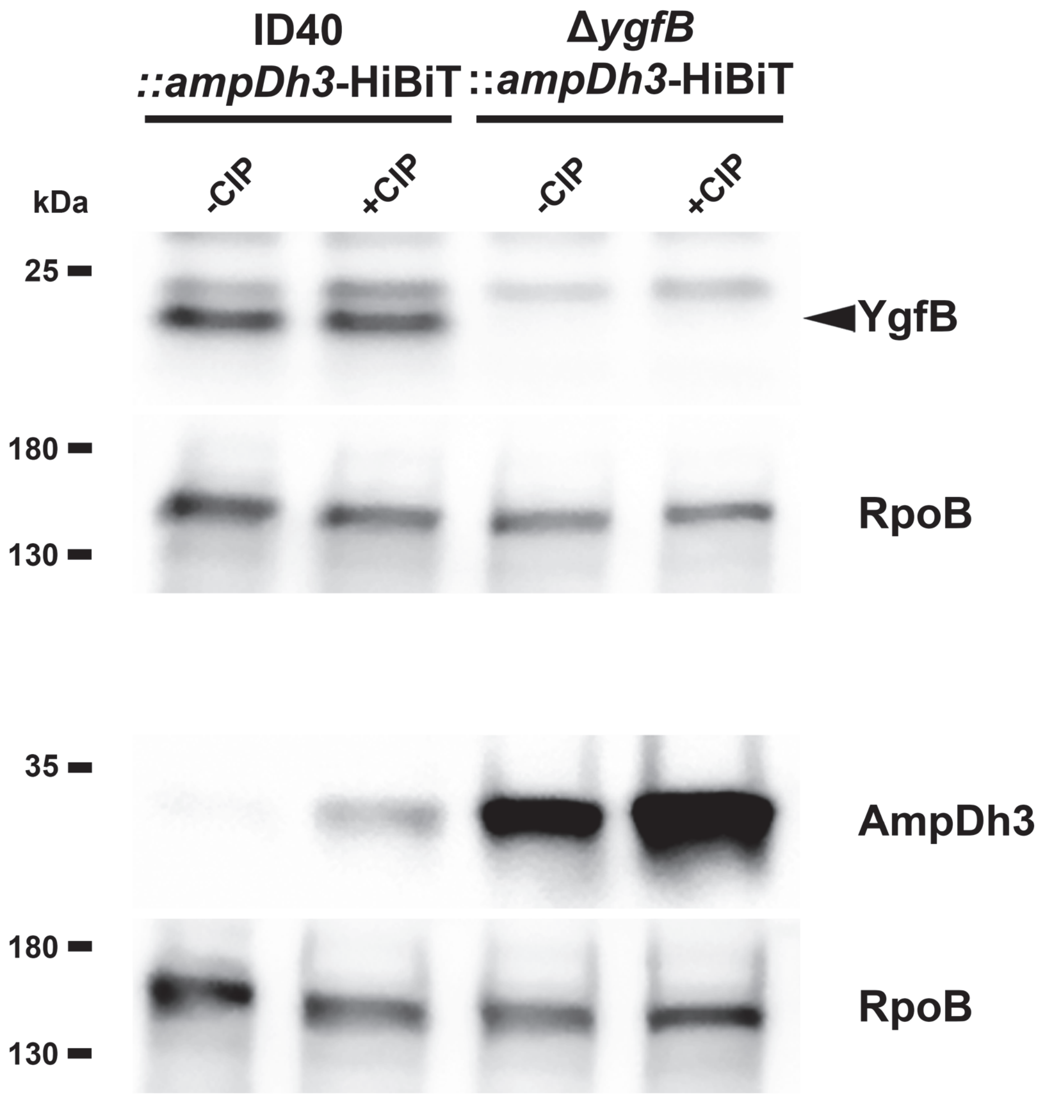

Supplementary Fig. 5: Production of AmpDh3 upon stimulation with subinhibitory concentrations of ciprofloxacin (CIP). The indicated strains were adjusted to a McFarland of 0.5 in LB medium and then treated with or without 2.5 µg/ml CIP for 18 h. Whole cells were lysed in Laemmli buffer. The detection of YgfB and AmpDh3 was done on separate Western blots, each with RpoB as a loading control. Protein detection was performed either using recombinant LgBiT and furimazine for AmpDh3-HiBiT or anti-RpoB and anti-YgfB, respectively, followed by HRP-conjugated secondary antibody and ECL as substrate. Blots shown are representative for three independent experiments.

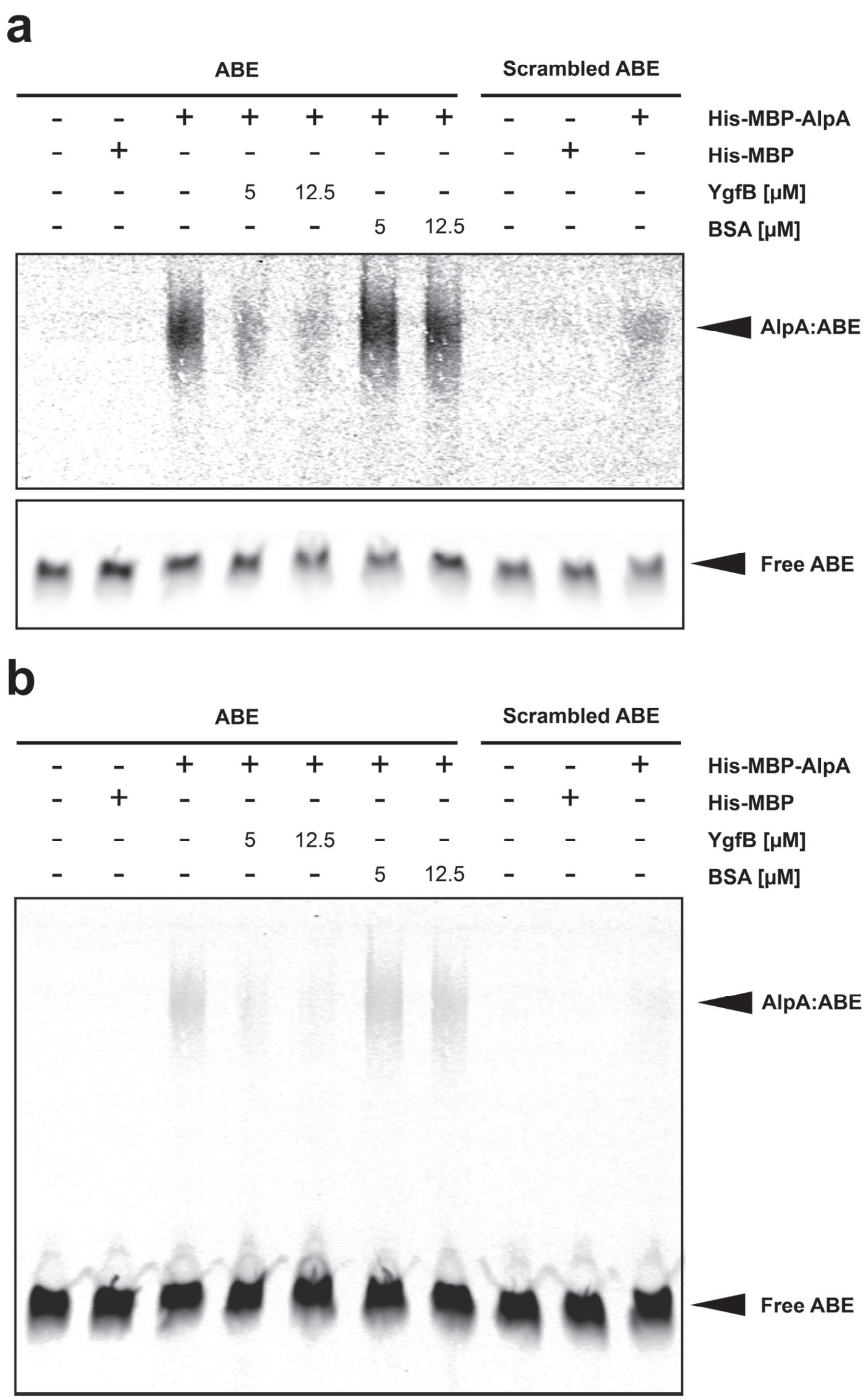

Supplementary Fig. 6: Interaction of AlpA with the AlpA binding element (ABE) of the *ampDh3* promoter. (a) EMSA using 0.3125 nM IRDye700-labeled AlpA binding element (ABE) or 0.3125 nM IRDye700-labeled scrambled DNA probe incubated with the indicated proteins. The assay shown here is representative of five experiments. Concentrations used: His-MBP-AlpA and His-MBP 1.25 µM; YgfB and BSA 5 µM and 12.5 µM. Detection of IRDye700 was performed using the Licor Odyssey detection system. The original image was converted to greyscale. Of note, to obtain a higher dynamic range the free dsDNA and the shifted DNA-protein complexes were recorded separately. (b) In this case fluorescence was recorded over the entire gel to simultaneously record free ABE and AlpA:ABE complex.

Supplementary Table 3: MIC values for the indicated strains (in mg/l). MEM meropenem, IMP imipenem, FEP cefepime, CAZ ceftazidime, PIP piperacillin, TZP tazobactam/piperacillin, AZT aztreonam, CIP ciprofloxacin. Green: reduced MIC compared to WT, Green bold: resistance is broken, nd not determined.

	**MEM**	**IMP**	**FEP**	**CAZ**	**PIP**	**TZP**	**AZT**	**CIP**
MIC S<	**2**	**4**	**8**	**8**	**16**	**16**	**16**	**0.5**
MIC R>	**8**	**4**	**8**	**8**	**16**	**16**	**16**	**0.5**
**ID143 WT**	32	32	16	32	>128	>128	>32	>4
**ID143Δ*ygfB***	2-4	**2**	**8**	**<2-2**	**<16**	**8**	**4**	>4
**ID72 WT**	16	32	32	>32	>128	>128	32	<0.125
**ID72Δ*ygfB***	**2**	**4**	**2**	**4**	**<16**	**<8**	**8**	0.125
**PAO1**	**<0.5**	8	**1**	**<1-1**	**<16**	**<4**	**2**	<0.125
**PAO1Δ*ygfB***	**<0.5**	**<1**	**1**	**<1-1**	**<16**	**<4**	**1**	<0.125
**PA14 WT**	**<0.5**	**<1**	**<1**	**2**	**<4**	nd	**4-8**	<0.125
**PA14Δ*ygfB***	**<0.5**	**<1**	**<1**	**2**	**<4**	nd	**4**	<0.125

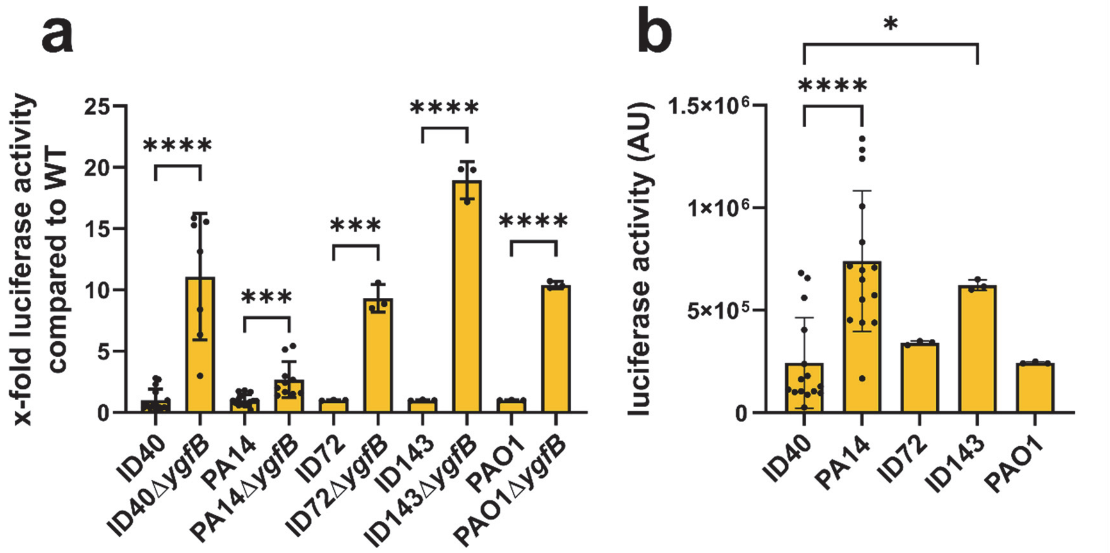

Supplementary Fig. 7: Impact of *ygfB* deletion on other *Pa* strains. (a) and (b) *ampDh3* promoter activity was determined as described in the materials and methods for the indicated strains using the plasmid pBBR harboring the reporter construct *ampDh3*-532-luc comprising the *ampDh3* promoter fragment between position -532 and -1 upstream of the CDS of Nanoluc. Data depict mean and SD. (a) Luciferase activity of *ygfB* deletion strains relative to the “wildtype” is shown using data from *n*=3-15 individual experiments. Asterisks indicate significant differences compared to the “wildtype” strain (*p<0.05, **p<0.1 ***p<0.001, ****p<0.0001; two-tailed Welch’s t-test)

(b) Basal promoter activity is shown for the indicated strains using data from *n*=3-15 individual experiments. Asterisks indicate significant differences compared to ID40 (*p<0.05, ****p<0.0001; one-way ANOVA, Dunnett’s multiple comparisons comparing to ID40). Source data are shown in **Supplementary Data 12**.

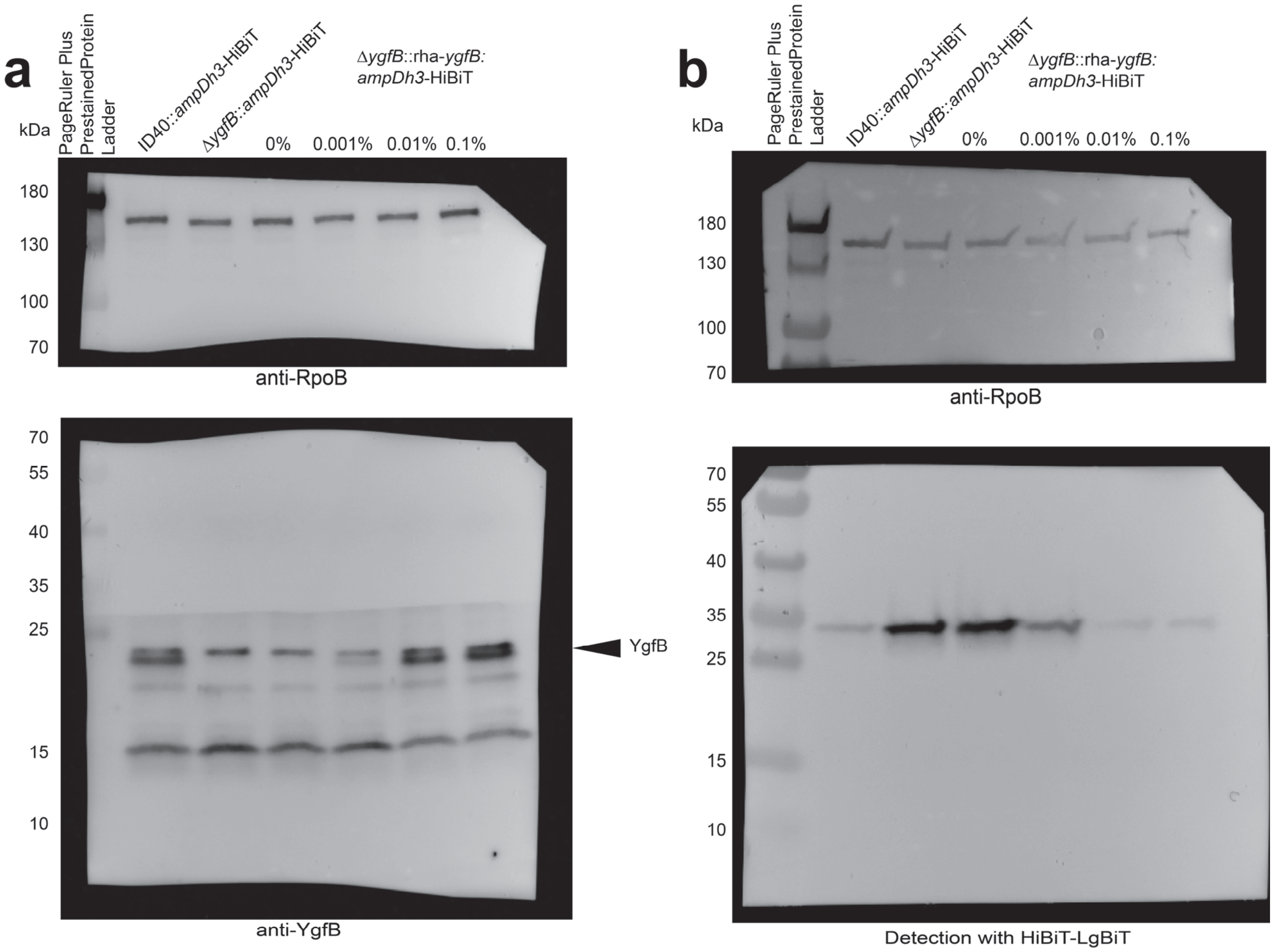

Supplementary Fig. 8: Uncropped Western blots shown in Fig. 1g. (a) Western blot detecting YgfB and RpoB as a loading control. To separately detect YgfB and RpoB on the same blot, the membrane has been cut apart horizontally at 70 kDa and each piece incubated with the respective antibody. Of note, due to the high unspecific binding of the YgfB antibody in order to obtain a better signal, the blot for YgfB has been covered above the 25 kDa mark during imaging. (b) Western blot detecting AmpDh3-HiBiT and RpoB as a loading control. To separately detect AmpDh3-HiBiT and RpoB on the same blot, the membrane has been cut apart horizontally at 70 kDa and each piece incubated with the respective antibody or NanoGlo HiBiT Blotting System.

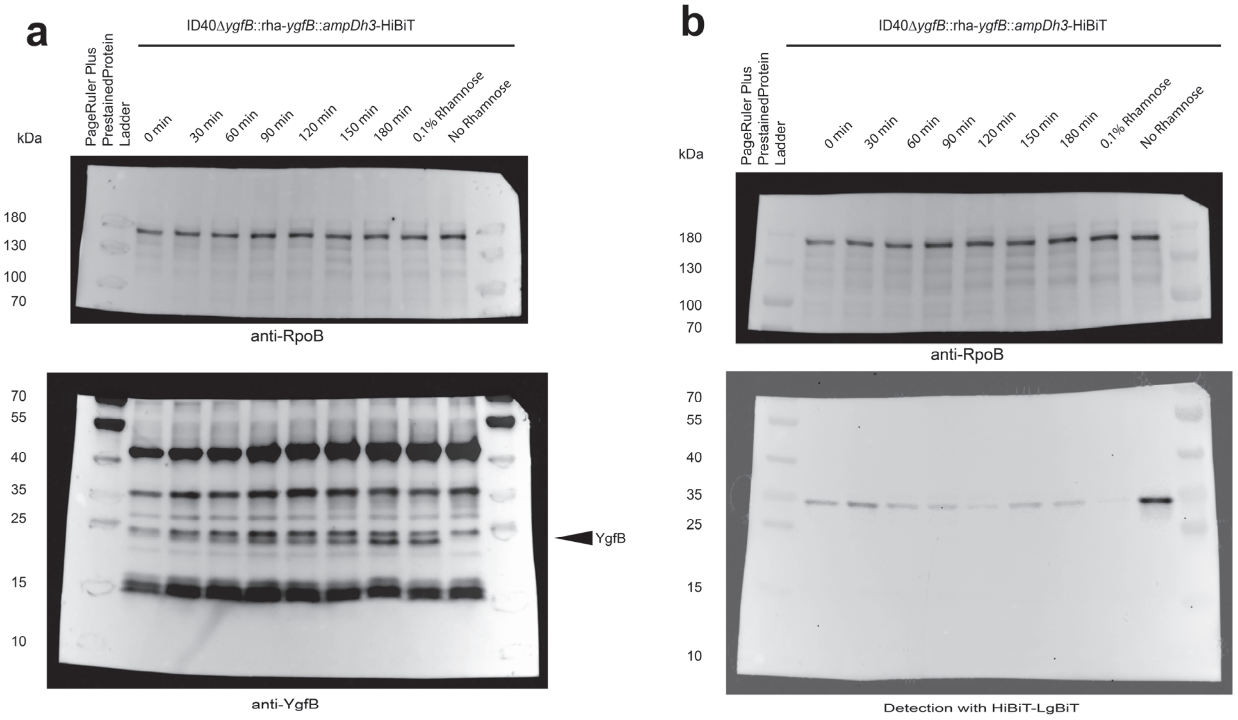

Supplementary Fig. 9: Uncropped Western blots shown in Fig. 2d. (a) Western blot detecting YgfB and RpoB as a loading control. To separately detect YgfB and RpoB on the same blot, the membrane has been cut apart horizontally at 70 kDa and each piece incubated with the respective antibody. (b) Western blot detecting AmpDh3-HiBiT and RpoB as a loading control. To separately detect AmpDh3-HiBiT and RpoB on the same blot, the membrane has been cut apart horizontally at 70 kDa and each piece incubated with the respective antibody or NanoGlo HiBiT Blotting System.

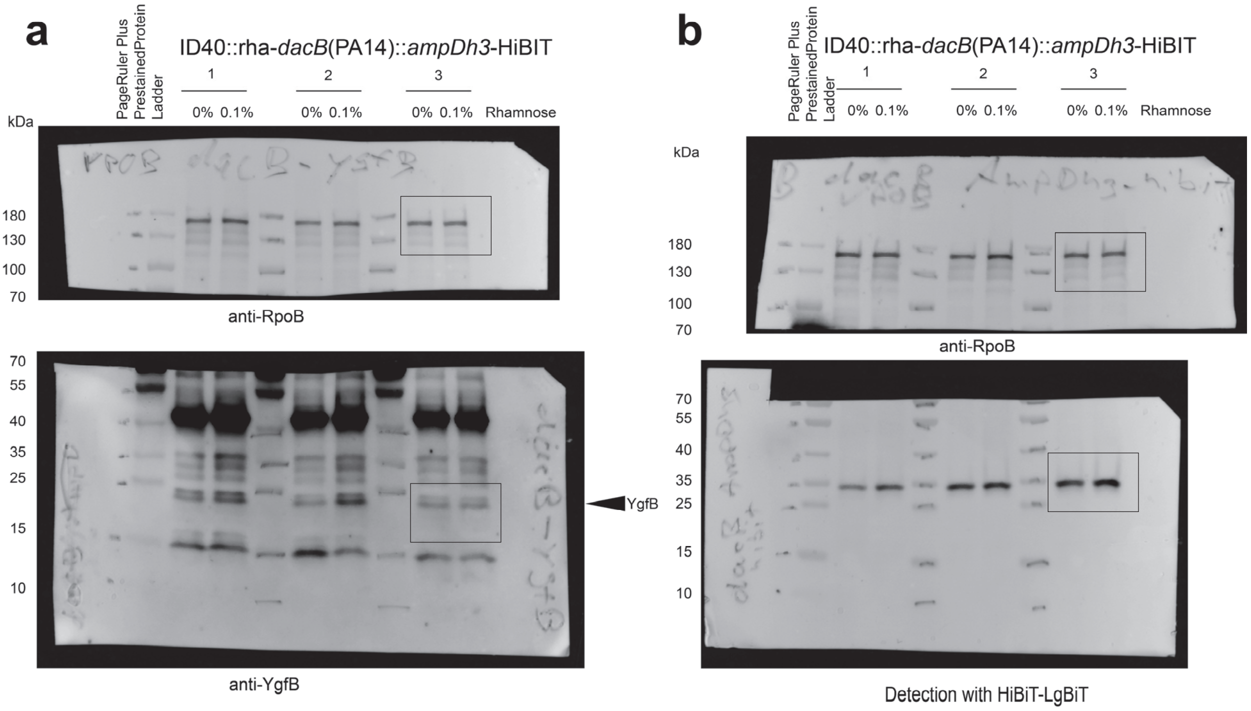

Supplementary Fig. 10: Uncropped Western blots shown in Fig. 5c. (a) Western blot detecting YgfB and RpoB as a loading control. To separately detect YgfB and RpoB on the same blot, the membrane has been cut apart horizontally at 70 kDa and each piece incubated with the respective antibody. (b) Western blot detecting AmpDh3-HiBiT and RpoB as a loading control. To separately detect AmpDh3-HiBiT and RpoB on the same blot, the membrane has been cut apart horizontally at 70 kDa and each piece incubated with the respective antibody or NanoGlo HiBiT Blotting System. Boxed bands are shown in Figure 5c.

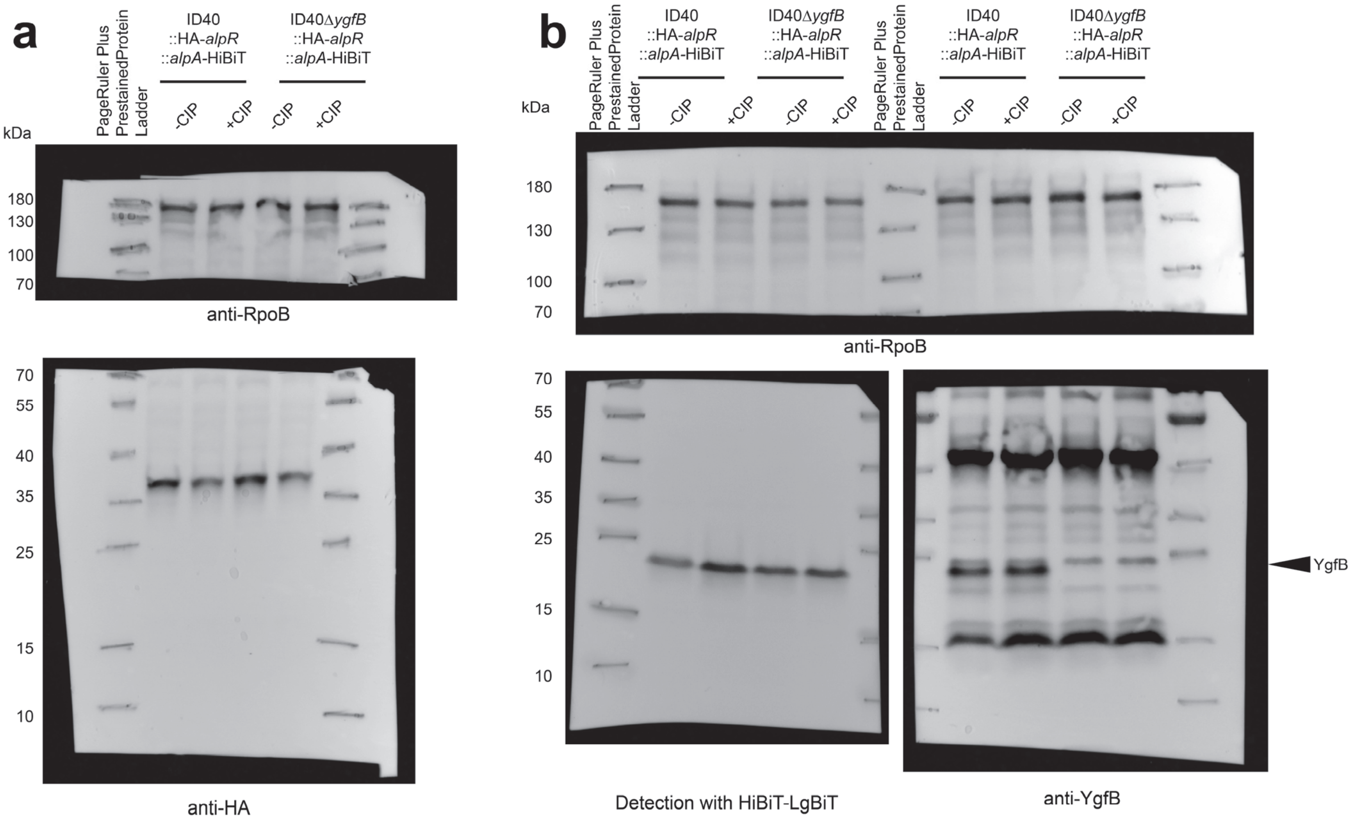

Supplementary Fig. 11: Uncropped Western blots shown in Fig. 7b. (a) Western blot detecting HA-tag and RpoB as a loading control. To separately detect YgfB and RpoB on the same blot, the membrane has been cut apart horizontally at 70 kDa and each piece incubated with the respective antibody. (b) Western blot detecting AlpA-HiBiT, YgfB and RpoB as a loading control. To separately detect AlpA-HiBiT YgfB and RpoB on the same blot, the membrane has been cut apart horizontally at 70 kDa as well as cut apart vertically on the line of the marker loaded in the middle. Each piece was then incubated with the respective antibody or NanoGlo HiBiT Blotting System.

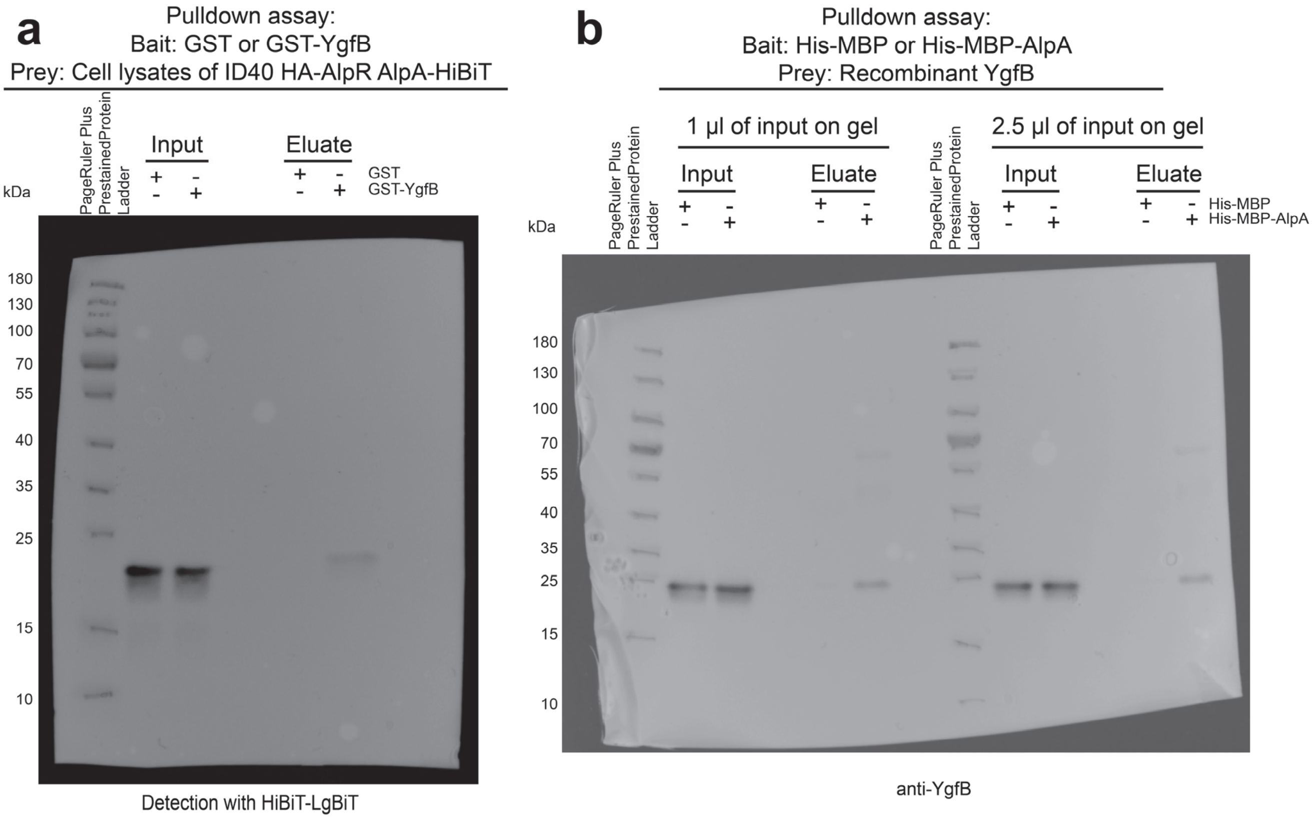

Supplementary Fig. 12: Uncropped Western blots shown in Fig. 8. (a) Western blot detecting AlpA. To detect HiBiT-labeled AlpA from whole cell lysates, the membrane was incubated with NanoGlo HiBiT Blotting System. (b) Western blot detecting YgfB. To detect YgfB the membrane was incubated with an anti-YgfB antibody. 1 µl and 2.5 µl of input sample were loaded onto separate lanes on the gel as indicated.

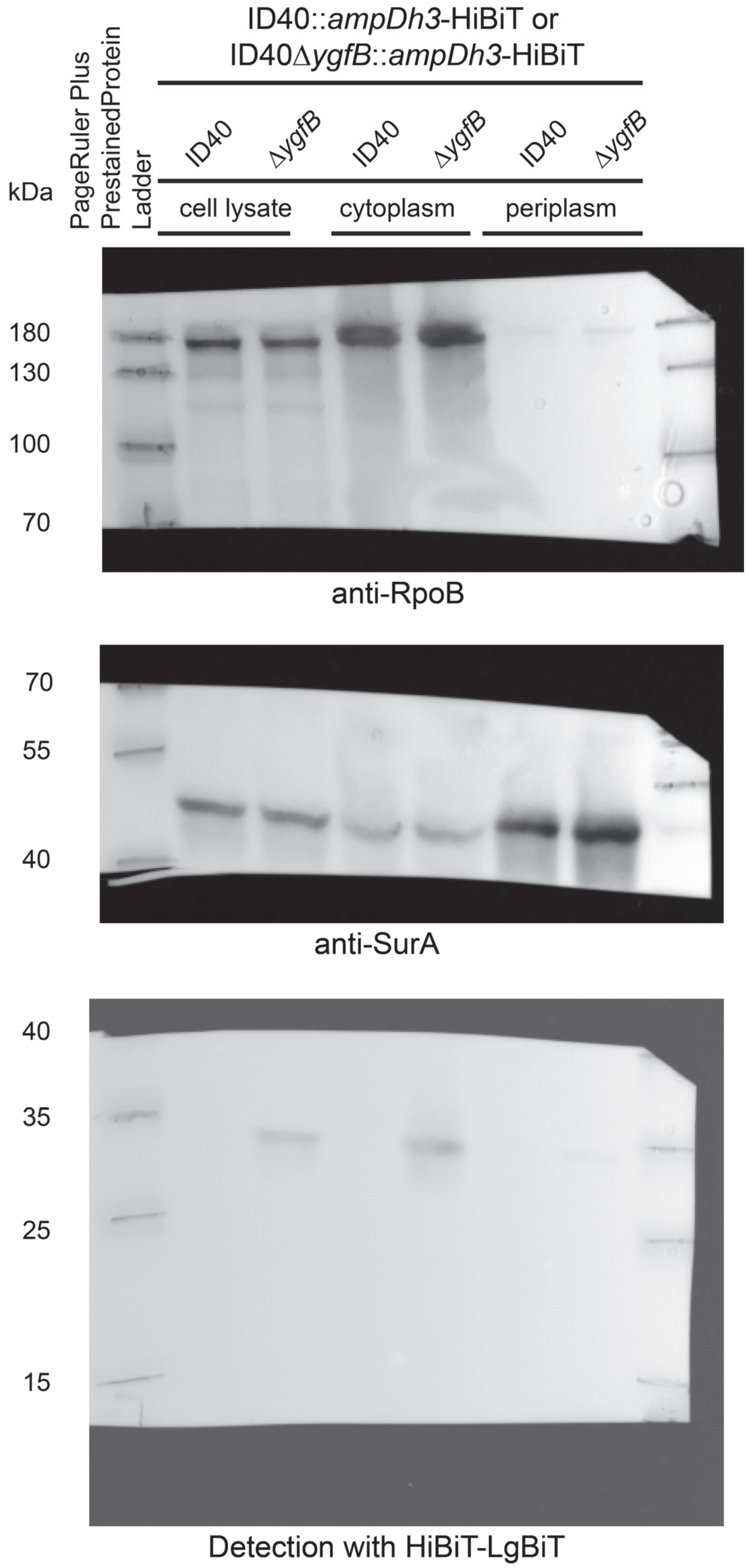

Supplementary Fig. 13: Uncropped Western blots shown in Supplementary Fig. S4. Western blot detecting SurA, RpoB and AmpDh3-HiBiT. To separately detect SurA, RpoB and AmpDh3-HiBiT on the same blot, the membrane has been cut apart horizontally at 70 kDa and at 40 kDa and each piece incubated with the respective antibody or NanoGlo HiBiT Blotting System.

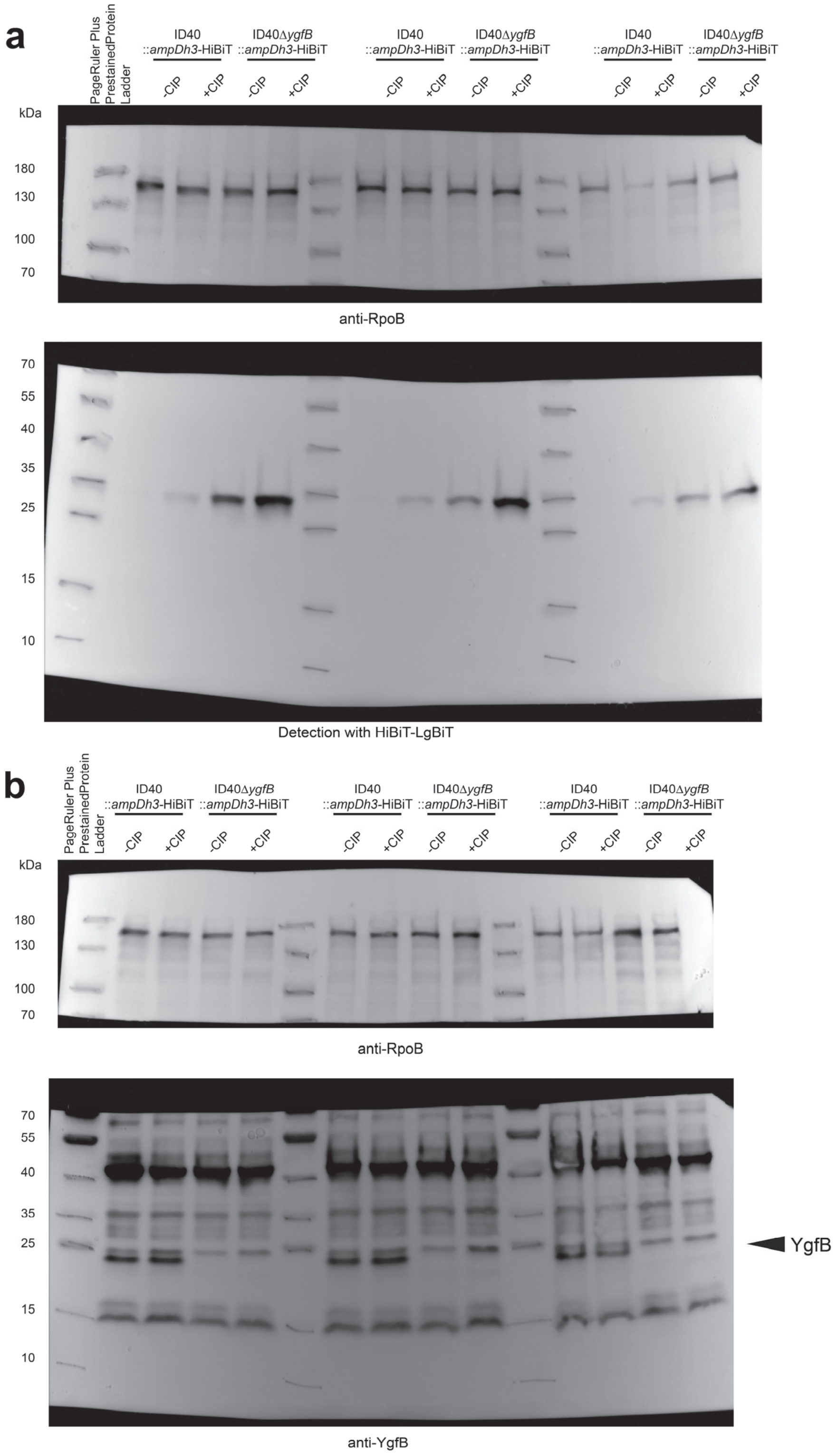

Supplementary Fig. 14: Uncropped Western blots shown in Supplementary Fig. S5. (a) Western blot detecting AmpDh3-HiBiT and RpoB as a loading control. To separately detect AmpDh3-HiBiT and RpoB on the same blot, the membrane has been cut apart horizontally at 70 kDa and each piece incubated with the respective antibody or NanoGlo HiBiT Blotting System. (b) Western blot detecting YgfB and RpoB as a loading control. To separately detect YgfB and RpoB on the same blot, the membrane has been cut apart horizontally at 70 kDa and each piece incubated with the respective antibody.

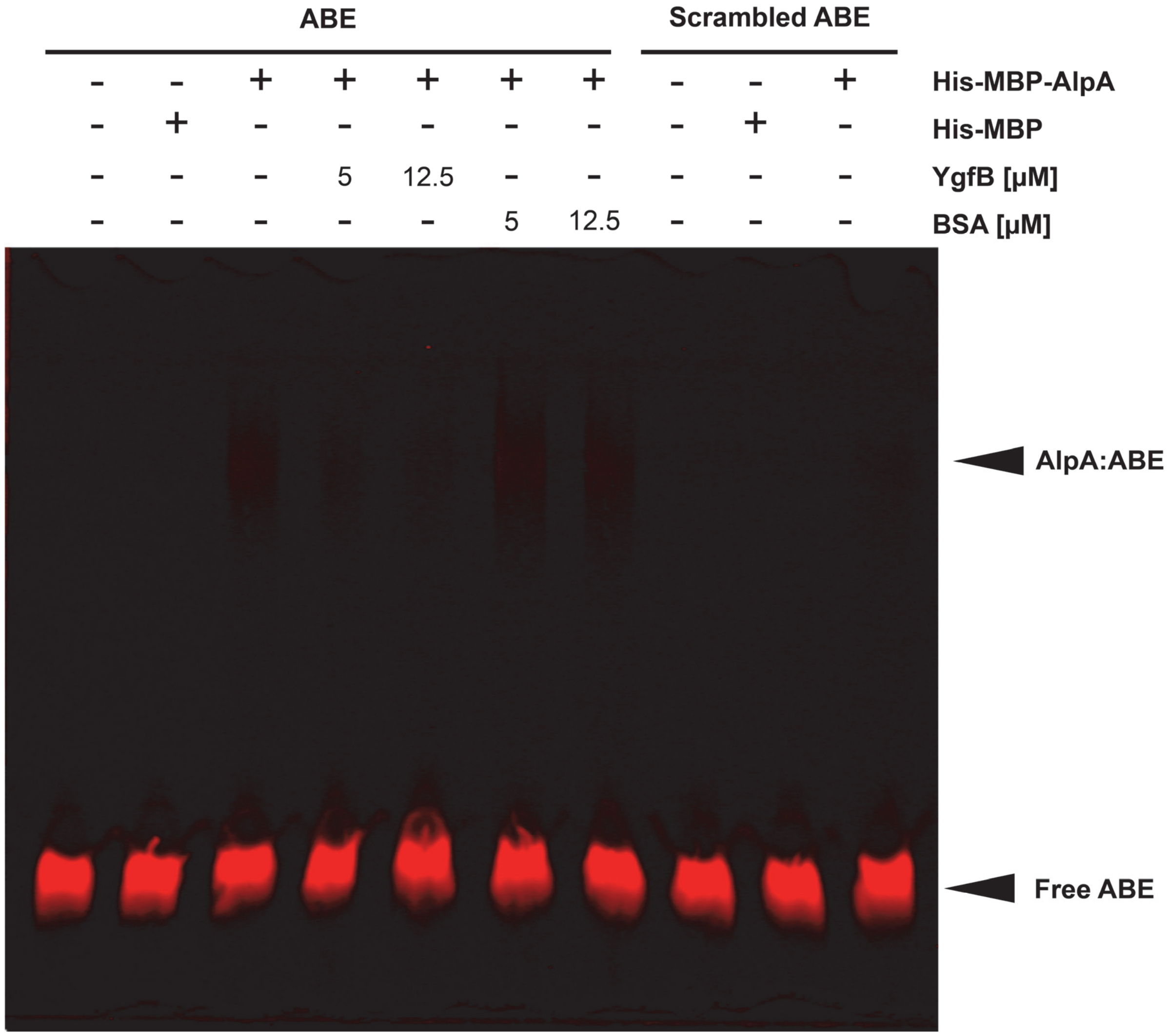

Supplementary Fig. 15: Uncropped and original image of EMSA shown in Supplementary Fig. S6.

Supplementary References

1. Sievers F*, et al.* Fast, scalable generation of high-quality protein multiple sequence alignments using Clustal Omega. *Mol Syst Biol* **7**, 539 (2011).

2. Zimmermann L*, et al.* A Completely Reimplemented MPI Bioinformatics Toolkit with a New HHpred Server at its Core. *Journal of molecular biology* **430**, 2237-2243 (2018).

3. Larkin MA*, et al.* Clustal W and Clustal X version 2.0. *Bioinformatics* **23**, 2947-2948 (2007).

4. Galkin A, Sarikaya E, Lehmann C, Howard A, Herzberg O. X-ray structure of HI0817 from *Haemophilus influenzae*: protein of unknown function with a novel fold. *Proteins* **57**, 874-877 (2004).

5. Crooks GE, Hon G, Chandonia JM, Brenner SE. WebLogo: a sequence logo generator. *Genome research* **14**, 1188-1190 (2004).

6. Schneider TD, Stephens RM. Sequence logos: a new way to display consensus sequences. *Nucleic Acids Res* **18**, 6097-6100 (1990).

7. Waterhouse AM, Procter JB, Martin DM, Clamp M, Barton GJ. Jalview Version 2--a multiple sequence alignment editor and analysis workbench. *Bioinformatics* **25**, 1189-1191 (2009).

8. Willmann M*, et al.* Multi-omics approach identifies novel pathogen-derived prognostic biomarkers in patients with Pseudomonas aeruginosa bloodstream infection. *bioRxiv*, 309898 (2018).

9. Sonnabend MS*, et al.* Identification of Drug Resistance Determinants in a Clinical Isolate of *Pseudomonas aeruginosa* by High-Density Transposon Mutagenesis. *Antimicrob Agents Chemother* **64**, (2020).

10. Klein K*, et al.* Deprivation of the Periplasmic Chaperone SurA Reduces Virulence and Restores Antibiotic Susceptibility of Multidrug-Resistant Pseudomonas aeruginosa. *Front Microbiol* **10**, 100 (2019).

11. Simon R, Priefer U, Puhler A. A Broad Host Range Mobilization System for Invivo Genetic-Engineering - Transposon Mutagenesis in Gram-Negative Bacteria. *Bio-Technol* **1**, 784-791 (1983).

12. Rietsch A, Vallet-Gely I, Dove SL, Mekalanos JJ. ExsE, a secreted regulator of type III secretion genes in Pseudomonas aeruginosa. *Proc Natl Acad Sci U S A* **102**, 8006-8011 (2005).

13. Meisner J, Goldberg JB. The *Escherichia coli* rhaSR-PrhaBAD Inducible Promoter System Allows Tightly Controlled Gene Expression over a Wide Range in *Pseudomonas aeruginosa*. *Appl Environ Microbiol* **82**, 6715-6727 (2016).

14. Choi KH*, et al.* Genetic tools for select-agent-compliant manipulation of *Burkholderia pseudomallei*. *Appl Environ Microbiol* **74**, 1064-1075 (2008).

15. Hoang TT, Kutchma AJ, Becher A, Schweizer HP. Integration-proficient plasmids for *Pseudomonas aeruginosa:* site-specific integration and use for engineering of reporter and expression strains. *Plasmid* **43**, 59-72 (2000).

16. Trebosc V*, et al.* A Novel Genome-Editing Platform for Drug-Resistant Acinetobacter baumannii Reveals an AdeR-Unrelated Tigecycline Resistance Mechanism. *Antimicrob Agents Chemother* **60**, 7263-7271 (2016).

17. Heeb S, Blumer C, Haas D. Regulatory RNA as mediator in GacA/RsmA-dependent global control of exoproduct formation in *Pseudomonas fluorescens* CHA0. *J Bacteriol* **184**, 1046-1056 (2002).

18. Heeb S*, et al.* Small, stable shuttle vectors based on the minimal pVS1 replicon for use in gram-negative, plant-associated bacteria. *Mol Plant Microbe Interact* **13**, 232-237 (2000).

19. Obranic S, Babic F, Maravic-Vlahovicek G. Improvement of pBBR1MCS plasmids, a very useful series of broad-host-range cloning vectors. *Plasmid* **70**, 263-267 (2013).
