# Supplementary material for: YgfB increases β-lactam resistance in Pseudomonas aeruginosa by counteracting AlpA-mediated ampDh3 expression
Source: Commun Biol. 2023 Mar 10;6:254. doi: 10.1038/s42003-023-04609-4 (PMC9998450; doi:10.1038/s42003-023-04609-4)
Supplement: Supplementary file 2 — Description of Additional Supplementary Files [file 42003_2023_4609_MOESM2_ESM.pdf]

Description of Additional Supplementary Files

File name: Supplementary Data 1
Description: Strains and plasmids used in this study.

File name: Supplementary Data 2
Description: Primers used in this study.

File name: Supplementary Data 3
Description: Source data used for Figure 1.

File name: Supplementary Data 4
Description: Source data used for Figure 2.

File name: Supplementary Data 5
Description: Source Data used for Figure 3.

File name: Supplementary Data 6
Description: Source data used for Figure 4.

File name: Supplementary Data 7
Description: Source Data used for Figure 5.

File name: Supplementary Data 8
Description: Source Data used for Figure 6 and Supplementary Table 2.

File name: Supplementary Data 9
Description: Source Data used for Figure 7.

File name: Supplementary Data 10
Description: Source Data used for Supplementary Figure 2.

File name: Supplementary Data 11
Description: Source Data used for Supplementary Figure 3.

File name: Supplementary Data 12
Description: Source Data used for Supplementary Figure 7.
