# Supplementary material for: YgfB increases β-lactam resistance in Pseudomonas aeruginosa by counteracting AlpA-mediated ampDh3 expression
Source: Commun Biol. 2023 Mar 10;6:254. doi: 10.1038/s42003-023-04609-4 (PMC9998450; doi:10.1038/s42003-023-04609-4)
Supplement: Supplementary file 15 — Reporting Summary [file 42003_2023_4609_MOESM15_ESM.pdf]

nature portfolio

Corresponding author(s): Erwin Bohn

Last updated by author(s): Feb 10, 2023

Reporting Summary

Nature Portfolio wishes to improve the reproducibility of the work that we publish. This form provides structure for consistency and transparency in reporting. For further information on Nature Portfolio policies, see our Editorial Policies and the Editorial Policy Checklist.

Statistics

For all statistical analyses, confirm that the following items are present in the figure legend, table legend, main text, or Methods section.

n/a	Confirmed	
☐	☒	The exact sample size (*n*) for each experimental group/condition, given as a discrete number and unit of measurement
☐	☒	A statement on whether measurements were taken from distinct samples or whether the same sample was measured repeatedly
☐	☒	The statistical test(s) used AND whether they are one- or two-sided<br>*Only common tests should be described solely by name; describe more complex techniques in the Methods section.*
☒	☐	A description of all covariates tested
☐	☒	A description of any assumptions or corrections, such as tests of normality and adjustment for multiple comparisons
☐	☒	A full description of the statistical parameters including central tendency (e.g. means) or other basic estimates (e.g. regression coefficient) AND variation (e.g. standard deviation) or associated estimates of uncertainty (e.g. confidence intervals)
☐	☒	For null hypothesis testing, the test statistic (e.g. F, t, r) with confidence intervals, effect sizes, degrees of freedom and P value noted<br>*Give P values as exact values whenever suitable.*
☒	☐	For Bayesian analysis, information on the choice of priors and Markov chain Monte Carlo settings
☒	☐	For hierarchical and complex designs, identification of the appropriate level for tests and full reporting of outcomes
☐	☒	Estimates of effect sizes (e.g. Cohen's d, Pearson's r), indicating how they were calculated

Our web collection on statistics for biologists contains articles on many of the points above.

Software and code

Policy information about availability of computer code

Data collection	- For imaging of the Western blots, the software of the Fusion Solo S imager (Vilber) was used<br>- For plate assays, the software of the Tecan Reader Infinite 200 Pro was used<br>- For detection of EMSAs using the Licor Odyssey imaging system, Image Studio Version 5.2.5 was used<br>- For RT-qPCR the software of the LightCycler 480 II was used
Data analysis	- Transcriptomic analysis: Mapping of sequencing reads and counting was performed using the subread package in R and the ID40 genome as a reference (https://www.ebi.ac.uk/ena/browser/view/LR700248). Differential gene expression analysis was performed using DeSeq2.<br>- Statistical analysis: Statistics were performed using GraphPad Prism 9.12 software

For manuscripts utilizing custom algorithms or software that are central to the research but not yet described in published literature, software must be made available to editors and reviewers. We strongly encourage code deposition in a community repository (e.g. GitHub). See the Nature Portfolio guidelines for submitting code & software for further information.

Data

Policy information about availability of data

All manuscripts must include a data availability statement. This statement should provide the following information, where applicable:

- Accession codes, unique identifiers, or web links for publicly available datasets
- A description of any restrictions on data availability
- For clinical datasets or third party data, please ensure that the statement adheres to our policy

Transcriptomic data generated in this study have been deposited with links to BioProject accession number PRJNA835697 in the NCBI BioProject database (https://www.ncbi.nlm.nih.gov/bioproject/PRJNA835697). Genomic DNA sequence of ID40 is available under https://www.ebi.ac.uk/ena/browser/view/LR700248. Primary data of experiments shown in the main text and the supplementary information are available as supplementary data. All other data as well as plasmids are available from the corresponding author upon reasonable request.

Human research participants

Policy information about studies involving human research participants and Sex and Gender in Research.

Reporting on sex and gender	No human research participants involved
Population characteristics	No human research participants involved
Recruitment	No human research participants involved
Ethics oversight	No human research participants involved

Note that full information on the approval of the study protocol must also be provided in the manuscript.

Field-specific reporting

Please select the one below that is the best fit for your research. If you are not sure, read the appropriate sections before making your selection.

☒ Life sciences ☐ Behavioural & social sciences ☐ Ecological, evolutionary & environmental sciences

For a reference copy of the document with all sections, see nature.com/documents/nr-reporting-summary-flat.pdf

Life sciences study design

All studies must disclose on these points even when the disclosure is negative.

Sample size	Sample sizes were chosen based on previous similar experimental settings which showed to deliver robust and reliable data
Data exclusions	No data were excluded
Replication	Data could be replicated by using biological replicates
Randomization	Due to the nature of the work with bacteria, randomization was not applicable
Blinding	Due to the nature of the work with bacteria, blinding was not applicable

Reporting for specific materials, systems and methods

We require information from authors about some types of materials, experimental systems and methods used in many studies. Here, indicate whether each material, system or method listed is relevant to your study. If you are not sure if a list item applies to your research, read the appropriate section before selecting a response.

Materials & experimental systems

n/a	Involved in the study	
☐	☒	Antibodies
☒	☐	Eukaryotic cell lines
☒	☐	Palaeontology and archaeology
☒	☐	Animals and other organisms
☒	☐	Clinical data
☒	☐	Dual use research of concern

Methods

n/a	Involved in the study	
☒	☐	ChIP-seq
☒	☐	Flow cytometry
☒	☐	MRI-based neuroimaging

Antibodies

Antibodies used

-Rabbit anti-YgfB: Manufactured by Eurogentec upon provision of recombinant GST-YgfB as described in Material and Methods
-Nano-Glo HiBiT Blotting System: Promega, Catalogue Number: N2410
-Rabbit anti-HA-Tag: CellSignaling; HA-Tag (C29F4) Rabbit mAb #3724
-Mouse anti-RpoB: BioLegend; Anti-E. coli RNA Polymerase β Antibody Mouse, Clone 8RB13, Monoclonal, Cat # 663903, RRID: AB_2564524
-Rabbit anti-SurA: Manufactured by Eurogentec upon provision of recombinant SurA as described in Material and Methods of Klein et al. 2019 doi:10.3389/fmicb.2019.00100
-Horseradish-peroxidase-conjugated goat anti-rabbit antibody: Dianova; Goat F(ab')2 anti-Rabbit IgG (H+L)-HRPO, MinX none, Polyclonal, Cat. Nr.: GtxRb-003-GHRPX
-Horse-radish-peroxidase-conjugated anti-mouse antibody: Invitrogen; Rabbit anti-Mouse IgG (H+L) Secondary Antibody, HRP, Polyclonal, Catalog # 61-6520, RRID: AB_2533933

Validation

-Rabbit anti-YgfB: Antibodies obtained from one rabbit were selected for best performance against ID40, the ID40ΔygfB deletion mutant and recombinant GST-YgfB, and subsequently affinity-purified against GST-YgfB protein. In the YgfB deletion mutant no band was observed for the expected MW when comparing to ID40 WT, while by complementing the strain with YgfB the band reappears.
-Nano-Glo HiBiT Blotting System: System was validated by Promega for use in western blotting as per their website
-Rabbit anti-HA-tag antibody was validated by CellSignaling for Western blotting as per their website
-Mouse anti-RpoB antibody was validated by BioLegend for Western blotting as per their website
-Rabbit anti-SurA antibody was validated by Klein et al. 2019 using deletion mutants and complementants (doi:10.3389/fmicb.2019.00100)
